# Supplementary material for: Bioinformatic Analysis Predicts a Novel Genetic Module Related to Triple Gene and Binary Movement Blocks of Plant Viruses: Tetra-Cistron Movement Block
Source: Biomolecules. 2022 Jun 21;12(7):861. doi: 10.3390/biom12070861 (PMC9313169; doi:10.3390/biom12070861)
Supplement: Supplementary file 1 [file biomolecules-12-00861-s001.zip › Fig_S1.pdf]

CCGGCCGACATTTATGTTGGACTTGATAGGGCGTATGTGTTTTGATTTATGCTTACTTACGGATGCCATCTTGTTAAGGTTTC
CTTTTGACGCTACGACTGCTGTCACGAAATCTTTTAGACAACAGGCGTCCCGGCAGTAAGCAAACGATCATTACCTTTCGCTT
ATCCGTTTCAGTTCGTAATGTCGGCCGGTAACTTTGCTCATGCTGTAATTACTGAAGGGTGGGCTTATTCTTCGGAATACGAA
AACGTTGGTGGTGCATTGGAGTCTGCACCATGGGTTCTTGCTGCTGAGTGGAAAGAGTTTCAGTTAACTTTATCTACTTTCAA
CTTTTCCATTCAGAGCGAACGCGACGCTATTCTGAAGGCGATTAAGAATCTTGATATTAGCTGCCCTTTCAGTTGGGAGGGTA
GGTTTCCTGCTCGTGAGTTTTGGGTCTGTTTGAAACCAGAACACACTGCATTGCTTCAACGTCTCGCTCACGCCGCCACTCAC
AAGGAAAAATACTCCGAAGTGCGTGAGTTGGCTCTTACAGGTGGTCGGGGTGCAGTTGCTGGTTCTTCTCTTCCTCCCCCTGA
TCGTGAGGCAGCGTATCCTGATAAGGGCGAGGCGTCCACCGCGTACTACAAAGGTTTGCAGGGTCTTGTGATTTCTGATACTT
GGCATCGAAGAAGTTTTGAGAAAGCAAACAAGTTGCGGTGGATACGGGATATCGAAGAGCCTGCTCCTGCTCCTTCTGGTGGA
GGAGGAGGTCGTTCGGGTGGTGGTGCTAGTGGTTCTGGTACTGGTGGTCGTGGTGATTCCAAGTCCTATATATTAGAGGACGA
CGGACGTGGCGACTAATGGCGTGGGTCGACCTTCCGTCTAGATATGTCGGCCTAGACGGAACGGTATGGGCTGTCAGTGTCAA
AGTGGACGAGAAACACCGACGTGTTAGAAACGTTGGGGTTGCGGATCCTTTACCTCGACATGAACACTTGGTGATCCCTTCCA
TGGCAATGAATTGCTATGGTTGGTTTGTGGTATTTACGGATTACGACACCGACGGCGTAGCTACTAGAGCTGTCGATGCAGGA
AGAGCTTTGAGAAGTGATATCTTAGCTTTGGGTGATCCGTACATATTCACAGACAGAGCTCTAGATGCTGCTAATGTGCAGAT
AGAGGTGCTGAGATCCGATGTGGTTAAGCTTGAGGATACGATACGACAGTTAGCATTAGCCGGTGGCGGTAACAATGTACAGT
TGTTAGAGGAACTTAAGGAAGCGCTGCATTCGTCTCGAGAAGAGTGTAGAACGCTGAGACATCAGGACGCTATCAAGACGGAA
GAATTGTTAAAATCGCAGTCAGAGGTTACCGCCCTGCGATCCGACTTGAATACGGTCCGTAATGACTTGTTTGCTTTACGGCG
ACATGCAAAGGATACCACCTTCATCTTTTCGAAGGATGGTCAGTTTTCTTTGCCTGACGCTAGGTCTTATGGGTGGAAGATAG
TTTGTAAAGAAGGAAAACCTTCTTGGGCTAGCACCAGGTATAAAGTTGAAGATGGTGTTCTGCTTGTGACACAGGAAGGAGAT
TTGATTGATGGGTACTGGTCTGACGGTAGGCCGCAGACTCTTTTAGCTAGGTTTGGAGCAAATTTTGAGATTTTGACTTGGAG
AGATGGTGTTATGTTTGATTTCGTGAACACTCCAGGTGGTGTTGTGTTCACTGTGTTGGGATACTCTCAACACTGGTGTTTGA
GAGCATAATGGTATGTTCAATAATGTAAATAAATAATTGCAAACATAAATACGATTCTCTGTAGTGATAGAATGTCTAGGTCT
TTTCTCGTTCTCGTTGTGTTGTGTCTTAGTTGTATCTTCCTAGGGTTGAGTGAAGGTAGAGTTGCTAAAACTGTACATTGTAG
GAATATTTTGCAGGAACTGACACAAGCCAAGGGTGGGTTGTTGCCAGTCTACTTTACGTACAGTTATCTAGCGGCCGACGGTG
GTATTTGCTGGGGTTCTTCTGTTTCTGCATTTGGTATCACAGAAAGAGCCCTTAATTACAAGAACAAGAAGGTTGCTGAATGC
TATTCTGCTAAACACTTGCTGCTTGTTCTCGGCGACTATGACTGCTTCTGTCCCTACGGCAACAGGTGTGTTTCTGAACTCTT
ATCTAAAAGAGACACCTAATGTCACAAGATACAATGTCGGGCCCATAAAATTTTCAGGGAGGAAGTTGGCCGACATATTGGAT
CGAAGTAGGGATTTCTTAGCTGACAGGTTGGATGAAATGTCGATGACCGACCCCTACTGGAATGATTGCTACGATGCCTTTAT
GGCCATGCCTTGTTCGTCAGGAAACGTTGTCAATATGATTGCTGTCACTGGAGGTCCAGGTTGTGGAAAGACCGAAGTGATAA
AGAGAATATGCAACGACGTGGATAATTGTACTGTCATGGTCCCCTTCAAGAGGCTGCAGACCGACTATCGGAATGGTGAGAGG
TGTTTTACACAAAACACTTGCGTTGCTAGGCACATGACCACCGATCTCCTCCTTGTCGATGAGTTCACAGGGTGTGACATTGG
TTTGGTATGTGCAGCAGCCCTGAATCAGAAGTGTTCCGAAGTTATTCTGTGGGGTGACATTATGCAGACTTGGCTGTCAGACT
CGGAAGGTGTGGGGTTTCGGGATTTCAAGATACCTTGTGCGGTAAAGTTGCGAAGCAATTATAGAAACCCATATGCGACGGTG
ACGCTATTGAACAGTATATTCTCAGACGACATGGTTTCTATGTCTGGGGATAAATCTTACGGCGTTATTGAGGTTAAACCGTT
GCATAATACGGTATTTATGGATGGTGTCGATTTGACCGGCACCAGAGAGAGTGTGGATGAGTTGAGGGATTGGTATATACCGG
CTGTTACGGTAAGGTCGTCGCAAGGAGCGACGTATGATGATGTGAATTTGTTGGTGTTTCAGAGAGATTTAAATATCTTCACA
AATTCATCGATCGTACGTGTGGCTCTAAGTCGTCATAGACGTAACCTTACCATATACACCGTCGATTGCCAGGCCTTCGAGAA
ACTAGTTACCACAGGCCGACTGGTAAACGACCACCTTGTGGGACTAGATTTGTATGAAATTGGGGCCAGTTCTGGTTGGTTTC
AATCTTGGCTTGTGTGGTTCAAATGTCGTCTTCTCGGGTTATAAATCAGAGGGCTGATCATACACCCACTTTGATAGCGGCTG
GAGCTGCCATTATTTTGGGTGGCTTAGTAGTGTGCTTGTTTGTTCTGAAGCCTCAAGAGAGATTTCACTCCGGGGGTGATCAC
GGTGTGGCAAGTTTTCGTAACGGAGGTCATTATTCTGACTCCAGAAAAGCTGTGTTTTCACCGGTGCATACGGTTCACAATCC
CAACGAGGGAAAATTATCCACTGATGCGCTGGTCACCGTGATAATACTGGTTTTGGCTCTCATAATAAAGTTTAAGAATGGCA
AGGATGCTAAGGGAGAGGACGCGAGCACATAGTTTTTATCCTTGGTTATGTGTTGCTTTTATTGTGTATATAGGTTGGCGTCT
TTATCCAAATACTGTTTCTGGAGGGAACGGACTGCCGGCCTTTGTTAATGGTGGTACCTGGGAAGATTCTAGAGAGATGACTT
TCTTTCCAGAAGGCACCAAATATAATGTTAGGGGAGATCCAATTATTCCGAACTCTTATAGGAGCGACGAATTGGAAGTGATA
CGCTCTTTTCTGAGCTGTGTTCACACATTGGTTCTCACCTTTATTTGTGGATTTTGGACCATTGTTGCGGTCATTTGTTATAA
AATATTACGTGCGTAAAGTGTGTAGTTGCAAACACGTCAGTGCAAGACGTTAAAAGAGCAATTTCTATTTGATGAGACAGTAT
CCGAGCAAAGACTCGTAAAGGTCTGAATCGTCAAACGCGAATGCGTTTCCTGTATGAAAAGGGAACGATTATTGCAACTCTGT
AGATAGAGTTTA(n)
