# Supplementary material for: Bioinformatic Analysis Predicts a Novel Genetic Module Related to Triple Gene and Binary Movement Blocks of Plant Viruses: Tetra-Cistron Movement Block
Source: Biomolecules. 2022 Jun 21;12(7):861. doi: 10.3390/biom12070861 (PMC9313169; doi:10.3390/biom12070861)
Supplement: Supplementary file 1 [file biomolecules-12-00861-s001.zip › Fig_S2.pdf]

ACTGTTTTACAGTGCTAAGTGGGGCTGTTTGGTTTTTTATTGCCGTAATTGGAAGGGCGTATAATTGGGTTTTTCTTACCGAC
ACAGTCGACTGTTGTCAATGTTTTTAAAACACAATTCACACACACCCCGAAACCTACTGGGATAATCCACCGGATCGCCATCC
CCTTGCGTGTAAGCAAAAGGTGAAGATTGAGGATAATTCGAAAACCATAAAACAGAAAACACAAAAGAAGAAGAAAGGAAGAA
AATACCGAACATGGCGAACAGAGAGTCATATACGCCATGGACAATGGACACCAGGATGTATGCCCTGCGAAAGTTCGTGAGGG
TATCAGACCTGGTGGCCGCCATTAACCACCTCAAAACGCTAGACTACGGTGTTGTGGCCAACCGTAGAGCGTTGAAATTAACA
CTATCGACCCTCGGGGACAGCGGAATCTTTAGCAGGTACACAAGGTTTCCCGGCGAAACAGACGATGGTTATGTGATGTACAT
GGACATGCAAGCCATGCCCTTGTCCACACACATCGAAAGTCTTCTAGCGGCGAGTGACACCAACGTGCCACTACCGAATTCGG
CGGCTCCTAACAACAACGGAAAGACCCCTGACGGGGCCGGTAGAGACCAGTCGAACGATTCGTCGTTCTCGATTCACAAAAAC
CTCAGCGCGATATCAATGCTGATGACCAATACCGTCTGTATAATAACACAAGAGACCTTTGAAATGGAAAAGCGGCTGACCTG
GGGTTAGCAATTAGCGGTGGCAGATCGAGCATCCTACAGGAACTACACGAGGAGGTTCGAGAAGGCCGTAGCCACCGCGGTCG
CGCTGAAGTACTTCCCCGGGACGAACCCGTCGAAGGAAGAACAGGAAATCTGGAACGCAGGAGAAGTACCGGCAGTTTACAGT
CGGACGGACGTTTTCTGGCCGAGAGAAGAAAACATCGTGCCACCTCCGTTGGTCGTTGCTGTGGAGTCTAGTTGGTTGATACC
GACTATGTGCTCCATAATAATAGCCGGCGGGTTGGTCATGATGGGTTTGAGCAGAAGGTACCACCTGGCCACCTACCTAAAGA
TGTATCTGAGGAACATGAGGGGAGAGTGGATCAGAAACAGGAACAACAAAAGGAGAGATGGCGGTGGCGATCCAGGGGCCCCC
CGGGTCACAGAGATGGAGAACATTCAGGAAAGAGAGACCCAGAGCCACCCAGGGCCAAAAACCACAGGATTCACAGGGATTGT
AGATCCCTACGAGGACATTGACGAGCCCCTGAACTGGGATAGTGACGAGGGCGTGGTCGAGTCGTACCTCTCCATGCTAACGC
AGTGCGGCATGTCGAGATTGGAGGTTTTGACCTTCTTACTCGACATGGACGTCGAGCTGTACGAGCTGGACCTGTCGTTCCTG
GAAATACTGGTGGAACAACACCCAGTTGCCACAACCGACCCCTTCGTCAGAGCACAACTAACGTCAGGAAGGATGGCACTGTG
GTCAGGACCGGCCACATCCTACATCCAGCTGATAAACTGTGAAACGCAGCATCAGCCTGGACCAGAGGAGCCCAAGGAAGAAA
GGAATGAGGAGACCTCAGACGTCCTCGTTGCGAGAATAAAAGCAGAGGTGGAAATGGAAAGGATAAGAGCGGATCTGGAGAAA
CACAGGTTGGAAGGGGAATGGTCCAGAGCAAACGCTCAACAAAAATATGAAGTCGCAGTGCCCGACATGAACGGAGGGTTCAA
ACTGGAAACAGCCTCGCTCTCACACCTACAGGCTATGAAACTGGCCTACGACCTGAGAGTCAGTTCGGCTGAGGAGGAAAAAC
TGAAAGCGTTGACCAGCAAGCCAAAAACCGAACAAGACTCTATGTCCATTGGGAAACATCCAAAGGAGATAGAGTTGCTGGAA
AACCAGATACTAGCCCAAAAGTCGGTCGTGGAGGCCAACAAAGTGGTGATGGCAAACTCGCAGACCCCCACAGAGAGGTGGAT
ACAATTGGGTATAAACACAGGGACCCAAATTGCGACGACCGCTGTGCAATGCGCGGTCCTCTACAGAGCCGTGGGGGCAATAA
ACACAGCGGGGGCAGTAGCATCGGCAGCAGAGAGTGTCCAAGGACACAGGCTGGGAGGACGACCCAGAATGATGAACATTTCA
GATCTGTAGAGTAGAGACCTTGTTAAAGTAGGAAGAACCGTAGCATTGTTTCAGGAGTTAATAGTATAAACACACAGTGACGA
ATATGGGAGATAGAGTAGTGAAACTGATAGAGAAAAGAGAAGCAAAGTTAGAAAAGGAAAGAGCTAGGAACCTAAAGCGTTTT
AGGATTGTAGAGGTAGAAAAGGGGGTGTGGTACCAGTTAGAGGAAGGAGAGTGTTTCTGTAGCAAATCAATACACAAACATTG
TGCGAAGTGTGGCGAACCGACGGACGGTTACTGCGTTATGTGCAGTTGCGAACTACGCGCAGAGCAAGCATACAGAACGCGTA
ACGAAAGAAAATGAAGGTTGCACGTACAGTTGCATTACTGGTTTTGGTACTGAGTACCCTAGGGCACGGGGCGGGGTCCACCG
AGTTAGATTGCAAATCAGCACTCCAGGTGCAAACCCAAAGGGCCTATGAGGACCTGCCCATCTACACCTCCAGGAGGGTGGGA
AATCTGTGGTTCAGCCGGGTGGACTGCTACCTAGGGAGCGCCTACGGAGTGGCCGGAAGAAAGAAGGTGGCGGACTGTCTGGC
AGCAGGAGTGCTGGTGAAAACCTGCCACTGCGATGGCCAAGCACTCAAAAGACGAAGTCTTTCAGATTGTGAGAAAGGCGATA
GGAGACACCCCAAACGTGGACTTCTTCCCCGAGTCCAGGCTGACATGGCAAGGTTGTTTTCTACAGTCTATACTGGAGAAAAC
AGCGGAGAACATGTTGGCCGATCTGGACAAAATGTCTGAGTCAGAGGCCTTCTACCCAATAACCAGGGAAGCCTACGAGAGGA
TACCCGAAGACGTTGGGAACAGAAAGATACACGTCTACAAAGTCGGGGGAGGACCAGGCTGTGGCAAGACCACCGTAATAAAG
AAGATATGCAAGGAGGAGGAATTATGCACCGTCATGGTTCCTTTTAAGAGGCTAAAGGCGGACTACACCGTGGAAAGATGTTT
TACGCAGCACACCTGCGTCGTCAGAGAAATGGCGACAAAGATACTACTGGTGGATGAATTCACCGGGTGCGATGTGGGTTTGG
TATGTGCGGCGGCGTTAAACCAAGGGTGCTCAAAGGTGATACTTTGGGGAGATGTCCTGCAGATTTGGCTGCAGGATGCAGAA
GGGCAAGGTTTCAAAGACTTTAAGATACCTGACTTTGCAGTAATGAGAGGCAATTACAGGAATCCAAGGTCCGATGTTCTGTT
ACTAAACTCACTCTTCGGAGACAACATGGCGCCGATGAAGAGTGGGGAGGGTGTGCAAGTCAGGGAACTTTTTGGGGATCCAC
TGGAGGAAGAAGGTGTGAACTTGACGTTCAGCAGACAGAGTGTTGATGAGTTGTCGCATCGGTATTTCATGGATGCCATTACT
GTTCGGTCTTCTCAAGGGGCTACTTACGATAAGGTCAATTTGTTTGTGTTCATGTGGGATTTAAATATGTTTACAAATGCGGC
TTTATTGAGGGTAGCTTTGAGTAGGCATAGGGTAAGGCTGGTGATATACACTTGTCACACACCATCTGCAAGGGACATAGTCG
AGACGGGTAGGATAACAAACGACCAACTGGTTGGCATAGATCACCACGAGATCAAAGCCTCTCTAGGTTTGGTAGCCACAATC
AAGAGACTACTAGGTTACTGATGTCTGGGAGGGTGTTGACGCAGAGGGCAGACTACACGCCCTTGTTTGTGATTGTTGGGGGC
GTCTTAATTCTGTCCGGGCTGTTTATGTTCAAAACGCCAGAGAGAATTCACTCCGGTGGAGATCAGGGGGTAGCTAGTTTCAG
GAACGGTGGAAGCTATACCGACGCGAGGAAGGCCGTCTTCAACCCGGTCCACAAAGTGCATGACCCAAGCAACTCAAAGGTCA
GCGCGGCAGGGGCCGAGGCGATTCTACTAGTCACCTCGGTGGTAATTTGTTTGATCCTAAAGTGGATAAATGGTCAGGGAGTA
CAAAAGAGAGACGACCTATAATGGGTTGTACATGGCGCTAATAGGGGTCCTATTGATTGTGGCGTACACAGTCAAACCGAGTT
TCGAACAAAAACACTCGGGGGGAAATGGCTTGCCAACGTTTGTGAATGGAGGGACATGGGAGAACACACAGACGCAAATAGCG
TTTCCTGAGGGGACAAGGTTTACCAAGGAGGGGAAGCCCGTATTCCCAGTCGATCGGTCGGCGGACGACATGATGTATATACA
CGTGTTAACCGAAATTGTGCAAGGAGTGAGGTTGATGTTCCGAACCATTGCACTCATGTGTGTGTTAGTTGGGTTTAGTCTTT
CGTGGAGAGTTATGAACTCTTTTTAAAGAGCTCAAATTCGTCATGTTCATGACGATAAAAGAAATAAATAAGACAGGCACAGC
CTGTGACAGAACTTTCGTTCTAGAAAGAAGGTGGAGACACCTTTCTTCGACAGTATCCGAGCAAAGACTCGTAAAGGTCTGAA
GGAGAAAAGGTTAATCTCCATCCCGTTTGAAAGGGGTGGGAAAAAGATTAGCGACCAGTATATTGGTCTA(n)
