# Supplementary material for: Bioinformatic Analysis Predicts a Novel Genetic Module Related to Triple Gene and Binary Movement Blocks of Plant Viruses: Tetra-Cistron Movement Block
Source: Biomolecules. 2022 Jun 21;12(7):861. doi: 10.3390/biom12070861 (PMC9313169; doi:10.3390/biom12070861)
Supplement: Supplementary file 1 [file biomolecules-12-00861-s001.zip › Fig_S3.pdf]

LHIDSGLPIAVTTVSRVRIFLSLVSRMASLVTETSVSLLDTSTVDINNMGDVDTVGSSSCVGSASISGLRSGSLGFGVGISDDAVLCDYTSVCF
TPYEISLFREQFAVTDAFIADARCYRALLGCFAKGTLARKILRDAFAECHGPLPIGLLYGLLDLISKAHSDNIGHLITLWSTPVYNLVTTGEHM
LHVDSSVRCINFFQLLMLVEDRCTQDLWVGMEAATMINMSMATNAANMASGHASKAFEAEVKATPKVSVILSTEEKRSLQRLIGFCPLFENKLA
VDNDHKVLYACRELARASYERASNCRNVNTKTLVVGSSWREVSNYISNPNIDHYFFCGDGKDMVRTTIKALEEAAKVKWKVMRASEKAEVRKRE
SLDTVPTGVGTVADGTPKKHQVDVFLGMKGLLKQAKEEGQVPGRFLFDVVNYTKDPSRVYSSLLFEDVGYNFSERNWLELFGMTNAMVGYGYMA
LPLELLFEDMPVTDKPVYRYNESTAWFCERSFLPEDGSSSFGRIRVTSASLSGNFTNGYEHPKDAWALMLRKPLWTHSSFSFALEVEITCRMGP
MVCFTITRRDYIGAVVRTIALPEDLEYVRLMDLVAISDRMKAKGSYTVCKPYPYFSARKAEVLALQSYLLSLDVKSLDLTNAMAYVRKMQNGAS
LVEAKLAPKWDLKGTDMPKFVVAVYHSTLLLRAKLYKDVDNIVCVEVNWKEKIKNILKRTANLVLEPMQFIWSWLYTSHLADQIILDMPNEHFQ
RFECGSNGPGGMSKLGRDMSFSPIFSEEFHIGEERKVFHPNHISRPELDVGPVAENQDDINQTTGHATAPNDRKGEVTYSNFDFEDSKFHFKGA
NPLLSFLKEGNKLRNDKDLHKSLLDFYNWRMEFDETTCSVCKALQGKTGAQIVECYHTGGPTVHNFSMTLTEVCDLRNKLKEDALKAPSGLAKT
FEDTHKILPVDAFSVEAEVEYIKGGPGCGKSYIIRALADPMLNLIAAPFLKLRSDYQQVEVEGRKVDFRFHTQHKACQETGSSTIFVDEFTALD
YQLLCVIVLRCGASKVVIVGDEQQTGILESAGEGINILNKIDVTCVNRHEPIVNFRNPAHTVRLLNYLYGYNMIPASDSNGKISFGSTLDFQES
SVKKDTTLIHYSWATHDILLCTDEDNKRDSKTTVRSNQGSTWENVILPITEYDSKLTLNDSLNIVALSRHKGNLDILLEGGDSCSPQVGTLRQM
IEGVPDVVNREDYVLRQFLGLETPRACAPKRRETMLQKEFRELVDLYDGFVPYDEEWPRQPTVFHKQEEMSNDDYTDIASSSTSDNVPDTIPQI
VEPIVTTDEEDCWSSQEEDKKPLLQGFEMAKLTNFPTPVRHTITSRPNNCLILALSEALPISRTDIDTKLRNGSHRSAAFYSRWLDSSQMSTIL
DVTAFAEFLDVRVSVKLINGDAIDDGHVIKCGPMSARDVMIVYDGNLKHYYHDKNVRPKRATFLDSLLKPSEKVARAVTERPIVEHVWKHGQDL
IDVLKFYDCNYATFAVLNDFLDVSGPKLFSGAVYKFLPSSEKLYGVDLRCGGATLTTSEVLTRVSDDLQLEDRDNFGLRQTYMDYVNEYIPRST
SSFLSAMVEDKGIVKEVRHVKPARDSYLLTGRVEYNSRQHYDLNFLNEETHTMVGNKFVTGTIDMGFMAPLNQRGHPKTNQVKYKALTTGPALV
YLKNSQWQTNHVLQERYLSTATSSMMSIAGVNTAKYIADLFVDECMDPNLDLVLDEETMNGLQLRAYGDMRIRNYQKQMNELDQAGANVCRFQL
KDIEKVAKDSINIAKAGQGIAAWSKVAQTKFMIPFRALNAALLKSLKPNVVYDNSYSEEEFVQRANVALNQTPSIAKNGVIDATACDAGQNAFT
QQIERYIIKRFGVSDEFLDRYFSFRSSYILQSDHVRAHVKNIKTSGEPGTLFGNSILMAALMNALLRGEGPWAMLMKGDDGVKRQANLQYNPVL
VKAIRDNCTLDFKLDIDVPMQFCGYALVSGMLVPCVERKLAKIATHKFKSYEHFKEYQESLRDWVNRVPTDPTLYAIFMSANAEVSGVSYSMVE
SMFEQIVSWSRVSGAQFELEFEDRVVIIDEQPIDYSLAAVDTKPMKFSTSWAPRELGKMVD-IPVQVLKLHGLNRCHHNCE-LLSQYYNII-
WSIDVNPDYLSDNSIRARLVKV-SGSFSKYDSRMKGC-FIAAL-IEF-K
